# Time to Recovery and Its Predictors among Children 6–59 Months Admitted with Severe Acute Malnutrition to East Amhara Hospitals, Northeast Ethiopia: A Multicenter Prospective Cohort Study

**DOI:** 10.1155/2020/5096201

**Published:** 2020-09-01

**Authors:** Telahun Kasa Tefera, Solomon Mekonnen Abebe, Melkamu Tamir Hunegnaw, Freezer Girma Mekasha

**Affiliations:** ^1^Department of Nursing, Dessie Health Science College, Dessie, Ethiopia; ^2^Department of Human Nutrition, Institute of Public Health, College of Medicine and Health Science, University of Gondar, Gondar, Ethiopia

## Abstract

**Background:**

Malnutrition has been among the most common public health problems in the world, especially in developing countries including Ethiopia. Even though the Ethiopian government launched stabilization centers in different hospitals, there are limited data on how long children will stay in treatment centers to recover from severe acute malnutrition. This study aimed to assess the time to recovery and its predictors among children 6–59 months with severe acute malnutrition admitted to public hospitals in East Amhara, Northeast Ethiopia.

**Methods:**

Institution-based, prospective cohort study was conducted in seven public hospitals in East Amhara and a total of 341 children were included in the study. The results were determined by Kaplan–Meier procedure, log-rank test, and Cox-regression to predict the time to recovery and to identify the predictors of recovery time. Variables having *P* value ≤0.2 during binary analysis were entered into multivarable Cox proportional hazards regression analysis. *P* value <0.05 was considered statistically significant.

**Results:**

The nutritional recovery rate was 6.9 per 100 person-days with a median nutritional recovery time of 11 days (an interquartile range of 6). The independent predictors like using NG tube for feeding (AHR = 0.44, 95% CI: 0.27–0.71), not entering phase 2 on day 10 (AHR = 0.19, 95% CI: 0.12–0.29), and being admitted to referral hospitals (AHR = 0.52 95% CI: 0.37–0.73) were associated with longer periods of nutritional recovery time.

**Conclusion:**

Both the recovery rate and the recovery time were within the acceptable minimum standards. But, special attention has to be given to children who failed to enter phase 2 on day 10, for those who needed NG tube for feeding, and for those admitted to referral hospitals during inpatient management.

## 1. Background

Malnutrition is a state in which the physiological functioning of the child's body is impaired due to either undernutrition or overnutrition [1]. It typically develops when the growth velocity and brain development are especially high during 6 to 18 months of age [2] and measured by stunting, wasting, and underweight [1].

Malnutrition can be acute or chronic while acute malnutrition is further classified into severe acute malnutrition (SAM) and moderate acute malnutrition (MAM) [3]. SAM is defined as very low weight for height (below -3z scores of the median World Health Organization (WHO) growth standards, or below 70% of the median of the National Centre for Health Statistics standard) and by the presence of nutritional edema [4]. In children 6–59 months of age, a middle-upper arm circumference (MUAC) less than 11.5 cm is also indicative of SAM [5].

SAM affects nearly 20 million under-five children; most of them are from Africa and Southeast Asia [2]. It is a significant factor in approximately one-third of the nearly 8 million deaths in children who are under 5 years of age worldwide [6]. Lower-middle income countries account for 76% of wasted children [7]. Children who are treated as inpatients because of complicated SAM have a reported mortality rate of 10 to 40% [8].

Due to weakened immunity, children with SAM are susceptible to infections [9]. These infections in turn result in reduced appetite and prevent the body's normal absorption of food which worsens malnutrition and further results in impaired growth and increased risk of death particularly when wasting is severe [5]. The risk of death among children with SAM is nine times higher than that for a healthy child [10]. Most deaths in children with acute malnutrition are related to infections such as malaria, diarrhea, pneumonia, tuberculosis (TB), and HIV/AIDS [11].

Health professionals unknowingly use practices that are highly dangerous for severely malnourished children [8, 11]. To solve such problems, WHO has developed guidelines for managing SAM [12] which was adopted later on to our context [13] and defined that inpatient management should include stabilization, transition, and phase 2 until full recovery at home.

According to the sphere standards, the recovery time of children hospitalized in stabilization centers should be less than four weeks and it is alarming if it takes longer than 6 weeks [14]. Even though sphere standards recommend shorter recovery times, most of the studies in Ethiopia showed that the median time to recovery was high and ranges from 14 days to 8.7 weeks [15, 16], respectively. But, children, due to various factors, do not get cured on time. By understanding this problem, the FMOH of Ethiopia established the National Nutrition Program (NNP) to improve the nutrition service delivery in health facilities [17]. Most of the studies were retrospective and also lack the most important variables such as the wealth and food security status of the families and institutional factors which could affect the recovery time of children with SAM [18, 19]. Therefore, this study tried to assess time to recovery and its predictors among children 6–59 months admitted with SAM prospectively.

## 2. Methods

### 2.1. Study Setting

The study was conducted from February 28 to May 02, 2019, in East Amhara Hospitals. There are a total of 34 hospitals in East Amhara, which provide services for an estimated population of 10 million. The study was conducted in seven public hospitals including Tefera Hailu Memorial Hospital of Sekota, Woldia Hospital, Mekaneselam Hospital, Hidar 11 hospitals, Dessie Referral Hospital, Kemise Hospital, and Debre Berhan Referral Hospital.

The aim of the study was to determine the time to recovery and its predictors among 6- to 59-months children with severe acute malnutrition who were admitted to East Amhara Hospitals. Regarding the settings of the study, the study was conducted in pediatrics wards of the selected hospitals where children were admitted. The children were admitted in separate rooms during phase 1 of their treatment and they shared rooms with other children during the transition and phase 2 of their treatment.

### 2.2. Study Design

An institution based prospective cohort study was conducted among children 6–59 months with SAM admitted to pediatrics wards of hospitals. The cases were followed for a maximum of 42 days. However, children who recovered earlier were only followed until recovery. Administration of the treatment was made by the frontline health workers, according to the national protocol without any direct intervention from the research team.

### 2.3. Study Participants

All children 6–59 months of age who were admitted with severe acute malnutrition were included in the study. According to the national protocol, patients fulfilling the admission criteria are enrolled and given a daily F75 during phase 1 and F-100 during the transition and phase 2 of the treatment.

### 2.4. Sample Size and Sampling Procedure

The sample size was calculated by using a sample size calculation for survival analysis in STATA 14 by taking the crude hazard ratio of 1.71, probability of withdrawals 0.4, probability of recovery rate 0.52 [18], alpha 0.05, and power of 80% which finally results in 325. By adding a 10% defaulter rate, the final sample size was 358. From 34 hospitals, 7 hospitals were selected by using a simple random sampling technique. The selected hospitals are Dessie Referral Hospital, Woldia Hospital, Kemise Hospital, Mekaneselam Hospital, Hidar 11 hospitals, Sekota Hospital, and Debre Berhan Referral Hospital. All children admitted to these hospitals were included in the study and those with congenital problems were excluded from the study.

### 2.5. Variables and Operational Definitions

The dependent variable of this study is time to recovery, and recovery time is defined as the number of days it takes from admission until a child is discharged after being claimed recovered from SAM [18, 20]. Recovery is reaching >80% of nutritional median weight for height and becoming free of acute infections within 42 days of treatment [20]. Death is a patient who died while he/she was being treated in the program in a facility [13], and defaulter is a SAM patient that was absent for two consecutive weighings [13]. A patient who could not meet the discharge criteria after six weeks of inpatient management is considered as a nonrespondent [13].

The independent variables considered in this study include sociodemographic variables: age and sex of the child, place of residence, age and sex of caretaker, marital status of caretaker, ethnicity, religion, educational status, and occupation; socioeconomic and household food security, routine medications, and complications: amoxicillin, vitamin A, ampicillin/gentamycin, folic acid, Albendazole/Mebendazole, pneumonia, diarrhea, HIV, TB, anemia, dermatosis, and CHF.

### 2.6. Data Collection Tools and Procedures

For data collection, two types of questionnaires were developed from various studies. The first was to collect the daily follow-up characteristics of children and it was adopted from the SAM management protocol. On the other hand, a structured questionnaire was developed in English after reviewing relevant literature and translated to the local language (Amharic) and translated back to the English language to check for its consistency. This questionnaire included the sociodemographic, food security, and economic status of patients' families/immediate caretakers.

Data were collected by interviewing the parents of children with SAM and by doing a physical examination and collecting laboratory investigations. The admitted children were followed for changes in weight, edema, and clinical signs like vomiting, diarrhea, cough, and convulsions which were assessed on a daily basis. In addition to this, MUAC was taken three times a week. Anthropometric measurements were by using standardized techniques and equipment.

Data were collected by fourteen trained health professionals (Clinical Nurses), and one B.S. Nurse was assigned as a supervisor for each hospital. Since the study was an observational study, the research team did not involve in any aspect of the treatment of the children with SAM. In order to ensure confidentiality and avoid data loss, all questionnaires were stored in locked cabinets throughout the study and accessed only by the principal investigator.

To assure quality, data collectors and supervisors were trained prior to data collection for one day regarding technique, ethics of data collection, and data collection process which was carried out by the principal investigator. A 5% pretest was conducted in Kobo Primary Hospital before the actual data collection. Data collectors, at the end of the data collection session, checked the questionnaires for completeness.

### 2.7. Data Processing and Analysis

Data were coded and entered using Epi-data Version 3.1 software. Then, they were exported to STATA version 14 for further statistical analysis. Both descriptive and analytic analyses were executed. The patient follow-up characteristics were described in terms of mean (standard deviation) and median (interquartile range) for continuous data and frequency distribution for categorical data. Frequency tables, graphs, and cross-tabulation were used to present the findings of the study. The results were determined using the Kaplan–Meier procedure, log-rank test, and Cox proportional hazard regression.

The food security status of each child's family was assessed based on FANTA's Household Food Insecurity Access Scale (HFIAS) indicators [21]. After checking assumptions, Principal Component Analysis was performed to determine the socioeconomic status of the families of children with SAM. Based on the PCA result, the families of children with SAM were categorized into three groups, namely, poor, medium, and rich.

Recovery time from SAM was estimated using the Kaplan–Meier procedure. A log-rank test was also used to test if there was a significant difference in recovery time between different groups of predictor variables. Those who were died, transferred out, and defaulted were considered as censored. Variables having *P* value ≤0.2 during binary analysis were entered into the multivariable analysis. *P* value <0.05 was considered as statistically significant for time to recovery. The Cox proportional hazard assumptions were checked by using Schoenfeld residuals and log (−log) S (*t*) plots. Intracluster correlation coefficients were also checked for individual and group level random effects. Both crude hazard ratio (CHR) and adjusted hazard ratio (AHR) with 95% confidence interval (CI) were computed to show the strength of association and to identify the main predictors determining the nutritional recovery time.

## 3. Results

### 3.1. Sociodemographic and Admission Characteristics

Of the total of 341 study subjects admitted to the hospitals, 181 (52.9%) of children were males while 85% of children were in the age group of 6–23 months with a mean age of 15.15 months (SD = ± 8.63 months). Correspondingly, from the parents, most of them (99.7%) were between the ages of 20 and 49, and 314 (92%) of them were married (Table 1).

### 3.2. Socioeconomic and Household Food Security Status

Of the total 341 families of children, 137 (40.18%) were poor, 111 (32.55%) were medium in socioeconomic status, and the rest 93 (27.27%) were rich. Regarding the household food security status of the families, 249 (73%) were food secure while the rest of them are food insecure. From families with food insecurity, 4 (1.17%), 80 (23.46%), and 8 (2.35%) were mildly, moderately, and severely food insecure.

### 3.3. Admission Characteristics

From a total of 341 children admitted with SAM, 281 (82.4%) were admitted with a nonedematous form of malnutrition. During admission, around 294 (86%) of them have MUAC less than 11.5 cm while 175 (51.32%) have WFH below 70%.

### 3.4. Comorbidities/Complications and Routine Medications

Pneumonia and diarrhea were the common comorbidities with a prevalence of 34.3% and 31.9% followed by anemia (18.8%). From routine medications, 307 (90%) of children with SAM received ampicillin/gentamycin. Likewise, from the study participants, 99 (29%) have taken amoxicillin while 31 (44.6%) from those who are eligible took deworming medications (Table 2).

### 3.5. Treatment Outcomes of Children with Severe Acute Malnutrition

**Figure 1:**
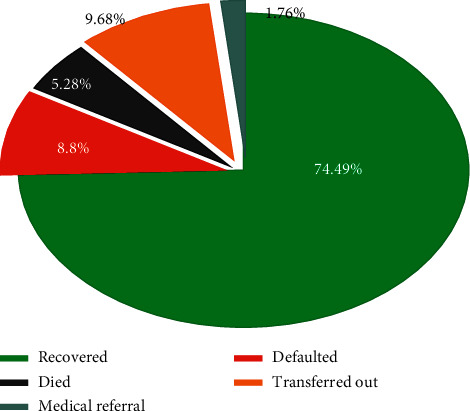
Treatment outcomes of 6–59 months children with severe acute malnutrition in East Amhara Hospitals, Ethiopia, 2019.

From the total study participants, 74.49% of them were recovered from SAM while 5.28% of them died (Figure 1).

### 3.6. Follow-Up Characteristics

From 58 children with edema, 12 (20.7%) failed to start to lose edema on day 4. On the other hand, from a total of 51 children, edema was present on day 10 in 10 (19.6%) of the cases. Eighty-seven children out of 341 (25.5%) failed to enter into phase 2 on day 10. In addition to this, from those children recovered from nonedematous SAM, 25.91% failed to gain more than 8 g/kg/day during phase 2. Thirty-one (11.15%) of them failed to gain more than 5 g/kg/d for 3 successive days.

### 3.7. Incidence of Recovery

The patients were followed for a minimum of 1 and a maximum of 42 days with 10 days [Q1, Q3, (9, 15)] median follow-up time. The overall nutritional recovery rate was 6.9 per 100 person-days (95 CI: 6.13–7.84) of observation. The incidence rate was found to be 6.8 (95% CI: 5.7, 8.0) and 7.13 (95% CI: 5.9–8.5) per 100 person-days in males and females, respectively. The death rate of children with SAM in this study was 2 per 100 person-days of observation.

### 3.8. Predictors of Time to Recovery

The predictors of time to recovery were failing to enter phase 2 on day 10, being admitted to referral hospitals, and using NG tube for feeding. The recovery time was delayed by 48% (AHR = 0.52; 95% CI: 0.37, 0.73) among children admitted to referral hospitals as compared with children admitted to general hospitals. The rate of time to recovery among children who did not enter phase 2 on day 10 delayed recovery time by 81% from SAM than their counterparts (AHR = 0.19; 95% CI: 0.12, 0.29). Children who used NG tube for feeding were 56% less likely to recover in shorter days than their counterparts (AHR = 0.44; 95% CI: 0.27, 0.71) (Table 3).

## 4. Discussion

This study tried to assess time to recovery and its associated factors among children 6–59 months with complicated, severe acute malnutrition admitted in public hospitals in East Amhara, Northeast Ethiopia. The nutritional recovery rate was 6.9 per 100 person-days with a median nutritional recovery time of 11 days (an interquartile range of 6). The independent predictors like using NG tube for feeding, not entering phase 2 on day 10, and being admitted to referral hospitals were associated with longer periods of nutritional recovery time.

This study revealed that the nutritional recovery rate is 74.49%, which was consistent with studies in Kenya [22] and South Wollo [23] and with the national minimum standards of the cure rate of 75% [24]. But, this recovery rate was better than findings from studies conducted in Ghana [25], Mekele [26], Gondar [27], Nekemte [28], and Bahir Dar [29] of Ethiopia. On the contrary, this rate is lower than studies conducted in Debre Markos and Finoteselam [20], Jimma [30], Wolaita [31], and Tigray [32]. This difference might be due to differences in settings, caseload, and severity of cases [33] and the availability of skilled and trained staff [34], socioeconomic status, and availability as well as the accessibility of therapeutic foods and medications [20].

The median nutritional recovery time which is 11 days in this study was similar to that in studies conducted in Debremarkos and Finoteselam [20], South Wollo [23], Gondar [27], Tigray region, and Northern Ethiopia [32] and the national minimum standards of the average length of stay [13]. However, it was longer than that in a study conducted in Zambia [35] and shorter when compared to studies conducted in Bahir Dar [18], Karat, and Fasha stabilization Centers in Southern Ethiopia [33] and Jimma [36]. These disparities might arise from differences in treatment and caring practices, health care settings, and other socioeconomic factors among the study areas [34].

The time to recovery among children who did not enter phase 2 on day 10 delayed by 81% from SAM compared to their counterparts. This was supported by a study conducted in Bahir Dar [18]. The reason might be due to severity, treatment environment, failure to complete the multichart correctly, insufficient staff, poorly trained staff, inaccurate weighing machine, and food prepared or given incorrectly. Unless children enter into the transition phase or phase 2, they will not be given therapeutic foods (F-100 and plumy nut) which can promote weight gain. Those formulas are designed for patients to rapidly gain weight. Therefore, children who enter phase 2 before the day of 10 will enjoy this advantage and recover early compared to their counterparts [12, 17, 18, 32].

Children who used NG tube for feeding were 56% less likely to recover in shorter days than their counterparts. This was in line with a study conducted in Geode Zone, SNNPR, Ethiopia [37]. This may be due to the reason that the NG tube is required when the child is not able to feed because of altered consciousness, shock or serious infection, failure of appetite test, and complications like aspiration [38].

In this study, the recovery time was delayed by 48% among children admitted to referral hospitals as compared with children admitted to general hospitals, which is consistent with studies conducted in North Shoa, Karat, and Fasha and Sidama Zones of Southern Ethiopia [32, 37, 38]. This might be due to the reason that referral hospitals are referral centers for primary and general hospitals and cases at referral hospitals are more complicated with increased severity, increased number of patients, and settings [32, 38].

### 4.1. Strengths and Limitations of the Study

Since it is a prospective cohort study, it has tried to address many important variables, including the sociodemographic and socioeconomic aspects of the families and the health facility-related factors. Each datum was collected before the outcome is known which enhances reducing bias and establishing stronger temporal relationships since the outcome is recovery. Even though this is a prospective cohort study, it has failed to assess the perception of caregivers on SAM.

## 5. Conclusion

Generally, the recovery rate and the nutritional recovery time of children with SAM in the study area were within the recommended national standards. Additionally, it was identified that being not able to enter phase 2 on day 10, using NG tube for therapeutic feeding, and being admitted to referral hospitals were associated with longer periods of recovery time.

The Regional Health Bureau must strengthen and follow closely the routine activities of screening children for malnutrition, which can assist in early diagnosis and management of children with SAM. Basic and refreshment training should be provided for hospital staff and program coordinators which can assist in improving the quality of nutritional therapy and adherence to the national standard protocol of SAM management. Special attention should be given for children who need NG tube for feeding, children who failed to enter phase 2 on day 10, and children admitted to referral hospitals while providing care to a severely malnourished child. Future researchers are recommended to assess the perception of parents/caregivers on SAM.

## Figures and Tables

**Table 1:** Sociodemographic status of the study participants in East Amhara, Ethiopia, 2019.

Variables	Categories	Frequency (*n*)	Percent (%)
Age of the child	6–11	134	39.3
	12–23	156	45.75
	24–35	40	11.73
	36–47	6	1.76
	48–59	5	1.47
Sex of the child	Male	180	52.79
	Female	161	47.21
Place of residence	Urban	69	20.23
	Rural	272	79.77
Age of caretaker	20–34	120	61.88
	35–49	180	37.83
	≥50	41	0.29
Sex of the caretaker	Male	15	4.40
	Female	326	95.60
Marital status of the caretaker	Married	314	92.08
	Widowed	16	94.13
	Separated	3	0.88
	Single	1	0.29
Ethnicity	Amhara	308	90.32
	Oromo	29	8.50
	Tigre	4	1.17
Religion	Orthodox	167	48.97
	Muslim	173	50.73
	Protestant	1	0.29
	Widowed	16	94.13
	Separated	3	0.88
Educational status of the caretaker	Illiterate	159	46.43
	Able to read/write	105	30.79
	Formal education (Grade)	73	21.41
Occupation	Housewife	201	58.94
	Farmer	83	24.34
	Gov't worker	29	8.50
	Others	28	8.21

**Table 2:** Routine medications and complications of children 6–59 months admitted with SAM to East Amhara Public Hospitals, 2019.

Characteristic	Category	Frequency (*n*)	Percent (%)
Amoxicillin	Yes	99	29.03
	No	242	70.97
Vitamin A	Yes	226	66.28
	No	115	33.72
Ampicillin/Gentamycin	Yes	310	90.09
	No	31	9.91
Folic acid	Yes	105	30.79
	No	236	69.21
Albendazole/Mebendazole	Yes	38	11.14
	No	303	88.86
Pneumonia	Yes	117	34.31
	No	224	65.69
Diarrhea	Yes	109	31.96
	No	232	68.04
HIV	Yes	29	8.50
	No	218	63.9
	Unknown	94	27.6
TB	Yes	10	2.93
	No	331	97.07
Anemia	Yes	64	18.77
	No	277	81.23
Dermatosis	Yes	21	6.16
	No	321	93.84
CHF	Yes	22	6.45
	No	319	93.55

**Table 3:** Predictors of nutritional recovery time among children 6–59 months with SAM managed to East Amhara Public Hospitals, North East Ethiopia, 2019.

Variables	Categories	Recovered	Not recovered	CHR (95% CI)	AHR (95% CI)
Educational status of caretaker	Illiterate	122	37	1	1
	Read/Write	76	29	1.38 (1.03, 1.85)	0.80 (0.56, 1.14)
	Formal education	56	21	1.81 (1.31, 2.5)	1.13 (0.76, 1.67)
Food security status	Food secure	183	66	2.24 (0.99, 5.06)	2.10 (0.80, 5.51)
	Mildly food insecure	3	1	3.89 (0.96, 15.7)	2.14 (0.39, 11.9)
	Moderately food insecure	62	18	1.1 (0.47, 2.53)	1.49 (0.54, 4.09)
	Severely food insecure	6	2	1	1
Still breastfeeding	Yes	208	72	1	1
	No	46	15	0.78 (0.57, 1.08)	1.04 (0.70, 1.55)
Appetite test at admission	Passed	76	14	1	1
	Failed	178	73	0.63 (0.48, 0.83)	0.82 (0.59, 1.15)
Pneumonia	Yes	87	30	0.69 (0.53, 0.89)	0.86 (0.61, 1.20)
	No	167	57	1	1
Diarrhea	Yes	81	28	1.48 (1.13, 1.94)	1.40 (1.02, 1.90)
	No	173	59	1	1
HIV	Yes	27	2	0.44 (0.29, 0.66)	0.73 (0.44, 1.22)
	No	227	85	1	1
Dermatosis	Yes	16	5	0.69 (0.41, 1.15)	0.39 (0.15, 1.05)
	No	238	82	1	1
Wealth status	Poor	96	41	1	1
	Medium	85	26	1.14 (0.85, 1.53)	0.80 (0.56, 1.14)
	Rich	73	20	1.32 (0.97, 1.79)	1.13 (0.76, 1.67)
TB	Yes	7	3	0.53 (0.25, 1.12)	0.78 (0.25, 2.45)
	No	247	84	1	1
NG tube for feeding	Yes	31	25	0.42 (0.29, 0.62)	0.44 (0.27, 0.71)^*∗*^
	No	223	62	1	1
Failure to enter PII on day 10	Yes	72	9	0.30 (0.22, 0.4)	0.19 (0.12, 0.29)^*∗*^
	No	182	78	1	1
Average weight gain	<8 g/kg/d	73	53	0.62 (0.45, 0.85)	0.83 (0.57, 1.22)
	>8 g/kg/d	181	34	1	1
Health facility	General hospital	145	53	1	1
	Referral hospital	109	34	0.83 (0.64, 1.06)	0.52 (0.37, 0.73)^*∗*^

^*∗*^Significant at *P* value of ≤0.05; HIV: human immunodeficiency virus; TB: tuberculosis; PII: phase 2.

## Data Availability

The datasets used and/or analyzed for the current study are available from the corresponding author on reasonable request.

## Abbreviations

HR: Hazard ratio
AHR: Adjusted hazard ratio
CI: Confidence interval
SAM: Severe acute malnutrition.
